# Arsenic Cancer Risk Confounder in Southwest Taiwan Data Set

**DOI:** 10.1289/ehp.8704

**Published:** 2006-01-13

**Authors:** Steven H. Lamm, Arnold Engel, Cecilia A. Penn, Rusan Chen, Manning Feinleib

**Affiliations:** 1 Johns Hopkins University Bloomberg School of Public Health, Baltimore, Maryland, USA; 2 Consultants in Epidemiology and Occupational Health LLC, Washington, DC, USA; 3 Georgetown University School of Medicine, Washington, DC, USA; 4 Georgetown University Graduate School, Washington, DC, USA

**Keywords:** arsenic, blackfoot disease, bladder cancer, cancer risk, confounder, dose–response relationship, southwest Taiwan, threshold model

## Abstract

Quantitative analysis for the risk of human cancer from the ingestion of inorganic arsenic has been based on the reported cancer mortality experience in the blackfoot disease (BFD)–endemic area of southwest Taiwan. Linear regression analysis shows that arsenic as the sole etiologic factor accounts for only 21% of the variance in the village standardized mortality ratios for bladder and lung cancer. A previous study had reported the influence of confounders (township, BFD prevalence, and artesian well dependency) qualitatively, but they have not been introduced into a quantitative assessment. In this six-township study, only three townships (2, 4, and 6) showed a significant positive dose–response relationship with arsenic exposure. The other three townships (0, 3, and 5) demonstrated significant bladder and lung cancer risks that were independent of arsenic exposure. The data for bladder and lung cancer mortality for townships 2, 4, and 6 fit an inverse linear regression model (*p* < 0.001) with an estimated threshold at 151 μg/L (95% confidence interval, 42 to 229 μg/L). Such a model is consistent with epidemiologic and toxicologic literature for bladder cancer. Exploration of the southwest Taiwan cancer mortality data set has clarified the dose–response relationship with arsenic exposure by separating out township as a confounding factor.

Southwest (SW) Taiwan has been the site for health studies for more than 45 years, initially because of the discovery of a unique peripheral vascular disease, blackfoot disease (BFD), that led to gangrenous amputation of the extremities and later because of the associations between high arsenic levels in local artesian well water and a variety of diseases, including cancers. A review of the history of these studies is useful as they have been used to estimate the carcinogenic risk from the ingestion of inorganic arsenic.

Epidemiologic studies on BFD were conducted by the Institute of Public Health of the National Taiwan University. All cases of BFD were found to have used the local artesian wells (Chen and Wu 1962), which were found to have high levels of arsenic (0.35–1.10 ppm) and algae (Chen et al. 1962). A high level of arsenic was suspected to be the most likely causal factor, although organic toxins from the algae were also considered (Chen et al. 1962), as later were fluorescent or humic substances (Lu 1990). BFD was particularly prevalent in the townships of Pei-men, Hsieh-chia, and Pu-tai (Chen and Wu 1962).

In 1965 a dermatologic survey was conducted in 37 villages in the BFD-endemic area, which found close associations between BFD, signs of chronic arsenicism (hyper-pigmentation and keratosis), and skin cancer (Tseng et al. 1968). This study showed a dose–response relationship for both BFD and skin cancer with respect to arsenic level, when well water arsenic level was stratified as < 0.30 ppm, 0.30–0.59 ppm, and ≥ 0.60 ppm (mg/L).

A death certificate study (1968–1982) was conducted on 84 villages in the BFD-endemic area that found a dose–response relationship between standardized mortality ratios (SMRs) of certain cancers and BFD prevalence rates of the villages and townships (Chen et al. 1985). The highest cancer rates were for the townships of Pei-men, Hsieh-chia, and Pu-tai, and the lowest for the township of I-chu. Cancer SMRs were greatest in the villages where only artesian wells were used as the drinking water.

This study was revamped in order to study arsenic levels as a risk factor. Because well water arsenic levels from the 1964–1966 survey (Kuo 1968) were known only for 27 of the 84 villages, the study was extended to include an additional 15 villages from the neighboring townships of Yen-shui and Hsia-in. The expanded study (Wu et al. 1989) also stratified villages by their median well water arsenic level as < 0.30, 0.30–0.59, and ≥ 0.60 ppm. The data of Wu et al. (1989) with the addition of an exposure stratum of median well water arsenic level < 0.1 ppm were used to calculate cancer potency indices (Chen et al. 1992). The potency indices for excess lifetime risk due to an intake of 10 μg/kg/day of arsenic were about 1.5 × 10^−2^ for male and female bladder cancers and lung cancers.

Morales et al. (2000) used the Wu et al. (1989) raw study data to model arsenic-attributed cancer risk, using village-specific census and mortality data and village-specific median well water arsenic levels rather than using the three-level arsenic strata methodology employed by Tseng et al. (1968) and Wu et al. (1989). Risks were calculated using a variety of models, either with SW Taiwan or all of Taiwan as the comparison population, and with no comparison population. The exposure–response curves showed a wide range of slopes at low-dose levels, depending upon choice of comparison population, model, and model parameters. Morales et al. (2000) performed a stratified SMR analysis using narrower strata than those of the Wu et al. (1989) study.

The U.S. Environmental Protection Agency (U.S. EPA 2001, 2005) and the National Research Council (NRC 1999a, 2001) have each used particular fits from the Morales et al. (2000) data set analysis as their basis for estimating the carcinogenic risk from the ingestion of inorganic arsenic. These estimates, like those of Morales et al. (2000), used the median village arsenic level as the sole explanatory variable for estimating the risk. The estimates also assumed a linear no-threshold model for extrapolation to low doses.

Previous analysis of the cancer mortality in the BFD-endemic area had shown township to be a discriminating factor for carcinogenic risk. Chen et al. (1985) stated that “the higher the BFD prevalence rate of a township, the greater the SMRs for cancers of bladder, kidney, skin, and lung of the township.” This article adds township as an explanatory variable in the quantitative analysis of the study by Wu et al. (1989).

We have expanded a data set of Wu et al. (1989) received from A. Schulman (U.S. EPA) and L. Ryan (Harvard University) by adding township and well water arsenic level information published by the NRC (1999b) and have examined the dose–response relationship for both the low-dose villages and high-dose villages with respect to possible explanatory variables, including township, apart from arsenic level (Table 1). Data can be obtained at StatLib website hosted by Carnegie Mellon University (Carnegie Mellon University 2006); click on “Get Data,” then search the term “arsenic.”

## Materials and Methods

Data underlying the study by Wu et al. (1989) are analyzed in this report. That study examined the 1973–1986 cancer mortality for the 42 study villages, based on death certificates that were coded to the *International Classification of Diseases: Manual of the International Statistical Classification of Diseases, Injuries, and Causes of Death, 8th Revision* (World Health Organization 1965), person-year distributions based on Taiwan household registration office data and in the data set of Morales et al. (2000) for study population and bladder and lung cancer deaths, and median village well water arsenic level as reported by the NRC (1999b). The outcome variables in the Morales et al. data set were limited to bladder and lung cancer mortality.

We performed linear regression analyses of the village data using Excel (Microsoft Corp., Redmond, WA) least-squares analysis with the village SMRs as dependent observations and with median village well water arsenic level as a continuous predictor. Approximate 95% confidence intervals (CIs) on *x*-intercepts from inverse linear regression were calculated. SMR = 100 was used as the reference value because it represents the SMR value at which the risk is not increased above that of the reference population. Stratified analyses have been based on township (individual or grouped), number of reported wells per village (one vs. multiple), artesian well dependency, and exposure strata (low vs. higher: < 0.13 ppm vs. > 0.25 ppm). The townships were identified by number by the NRC (1999b). The number of wells in each village was also identified by the NRC (1999b). Twenty villages had only one reported well, 10 had two wells, 7 had three to five wells, and 5 had six or more wells. Questions of measurement error or exposure misassignment have been raised, either because of the wide range of measurements that might be behind a median (Morales et al. 1999) or because the measured well may not be the actual well of use (Brown and Chen 1995). Well multiplicity was examined as a surrogate for distinguishing between those villages that entered the analysis on the basis of a “median” village well water arsenic level (i.e., those villages with multiple wells) and those villages whose exposure assignment was not based on a median (i.e., single-well villages). A village was operationally defined as being “artesian well dependent” if all its wells had arsenic levels > 0.325 ppm, based on the observation of Chen et al. (1962) of arsenic levels in artesian wells in the endemic area, which was cited by both Chen et al. (1985) and Wu et al. (1989). The definition of “low-dose village exposure” was based on the gap in the median village well water arsenic levels between 0.13 and 0.25 ppm and the observation by Wu et al. (1989) that arsenic content of well water samples had two clusters, with the low-dose cluster < 0.25 ppm.

We calculated SMRs for individuals 20 or more years of age for individual villages or groups of villages, with SW Taiwan data used for the reference population. We also calculated confidence intervals on SMRs from a Poisson distribution on the basis of the observed number of cancer deaths. Analysis of variance (ANOVA), regression analyses, and hierarchical regression analyses have been performed using SAS software (SAS Institute, Cary, NC).

## Results

**Figure 1:**
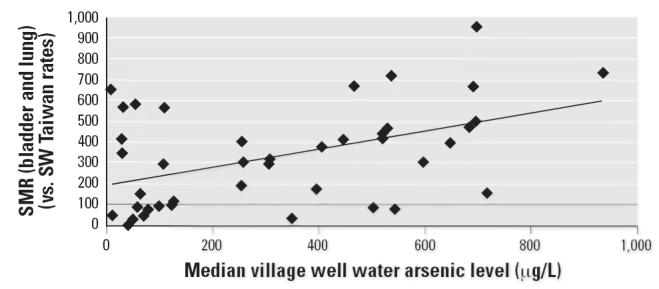
Bladder and lung cancer (combined) SMRs for the 42-study villages by median village well water arsenic level (μg/L). *y* = 0.429*x* + 189; *R*^2^ = 0.21; *p* = 0.03.

The present analysis is based on the 1973–1986 bladder and lung cancer mortality experience of the 42 villages studied by Wu et al. (1989)—27 from the study by Chen et al. (1985) of the four BFD-endemic townships and 15 from two neighboring townships. In total, this comprises 478,776 person-years of observation (≥ 20 years of age) and 181 bladder and 268 lung cancer deaths (449 total deaths). The primary exposure variable is the median village well water arsenic level that represents one well for 20 villages and multiple wells (range, 2–47) for the other 22 villages. Overall, linear regression analysis of the 42-village bladder and lung cancer SMR data on the median village well artesian level (parts per million) showed an explanatory model that accounted for only 21% of the variability (*p* = 0.03) (Figure 1). The *y*-intercept is at SMR = 189, above the no-increased risk line at SMR = 100.

A number of investigators have considered various alternative sources of variability in order to better understand the data. For example, the median village well water level may to some degree misrepresent the village-specific exposure because it ignores the marked variability in well water arsenic measures for a number of villages. The extreme example is that of village G in township 0, which has a range of well water arsenic measurements from 0.010 to 0.770 ppm but enters the analysis with a median of 0.030 ppm. Chen et al. (1985), the study that provided data for two-thirds of these villages (27/42 = 64%), pointed out that township, BFD prevalence, and artesian well dependency were each significant determinants of the cancer risk. Lamm et al. (2003) demonstrated that artesian well dependency was at least as strong a determinant of bladder cancer risk in this data set as was median village well water arsenic level.

We have used the village-specific data that are available for the villages studied by Wu et al. (1989) to examine singly and collectively the various hypothesized alternative sources of variability in the regression of cancer mortality risk (bladder and lung) on arsenic exposure level (median village well water arsenic level). Unfortunately, village-specific BFD prevalence ratios are not in the available data.

The NRC data (NRC 1999b) allowed us to examine three dichotomous potential sources of variability—well multiplicity, artesian well dependency, and exposure strata (low dose vs. higher dose)—as well as township. Each of the dichotomous variables was found to be a predictive factor for bladder and lung cancers in the one-way ANOVA, with *p*-values of 0.01–0.02.

We have looked at township as a stratifying variable and examined the dose response between bladder and lung cancer and median village well water arsenic level for each of the six showed a statistically significant association with *p*-values of 0.019, 0.011, and 0.019, respectively. Townships 0 and 3 did not show significant association, with *p*-values of 0.24 and 0.91, respectively. As township 5 had only two villages in the study, the *p*-value is undefined. The townships were grouped into those that showed a significant dose–response relationship with the arsenic exposure (townships 2, 4, and 6) and those that did not (townships 0, 3, and 5). Township group (as a dichotomous variable) was also a significant determinant of risk (*p* = 0.006) in a one-way ANOVA analysis.

Stepwise regression analysis demonstrated that median village well water arsenic level (parts per million) and township group were the only significant factors. The inclusion of either well multiplicity or artesian well dependency did not significantly increase the explanatory power of the model. The same result was found for bladder and lung cancer individually and combined and by sex individually and combined. The single exception was that the mortality risk for female lung cancer was best explained by township group and well multiplicity but not by median arsenic level.

**Figure 2:**
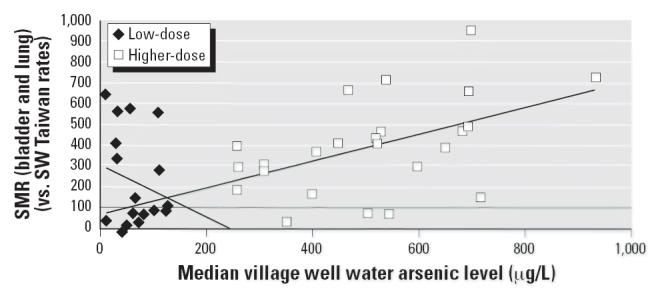
Bladder and lung cancer (combined) SMRs for low-dose villages and for higher-dose villages by median village well water arsenic level (μg/L). Low-dose villages (*n* = 18): *y* = 1.275*x* + 312; *R*^2^ = 0.04; *p* = 0.42. Higher-dose villages (*n* = 24): *y* = 0.639*x* + 71.2; *R*^2^ = 0.24; *p* = 0.02.

The combined bladder and lung cancer mortality was significantly higher in the higher-dose villages (SMR = 402.5; 95% CI, 358 to 447) than in the low-dose villages (SMR = 178.5; 95% CI, 148 to 209). The dose–response relationship was examined within each subgroup (i.e., the low-dose and higher-dose villages), with the expectation that the slope of the dose–response relationship for the higher-exposure villages and the slope of the dose–response for the low-dose exposure villages would be similar (Figure 2). However, the regression lines for the linear regression analysis of the cancer risk against median village well water arsenic level (parts per million) for the low-dose (*n* = 18) and higher-dose villages (*n* = 24) were dissimilar. The higher-dose villages showed a significant positive dose–response relationship for bladder and lung cancer SMR (*n* = 24; *R*^2^ = 0.24; *p* = 0.02), whereas the low-dose villages showed a nonsignificant negative dose response for bladder and lung cancer SMR (*n* = 18; *R*^2^ = 0.04; *p* = 0.42).

**Figure 3:**
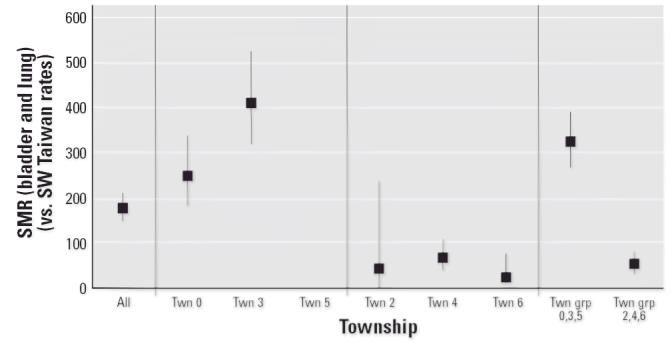
Bladder and lung cancer (combined) SMR (± 95% CI) for low-dose villages by township (Twn).

The data for the low-dose villages were then examined to seek a basis for the negative slope. Township was examined as a potential source of cancer risk variability for the low-dose villages. For instance, the five low-dose range villages in township 3 had median well water arsenic levels of 10, 32, 32, 56, and 65 ppb (μg/L), with bladder and lung cancer SMRs of 649, 348, 568, 587, and 154, respectively, whereas the five low-dose range villages in township 4 had median village well water arsenic levels of 42, 60, 80, 123, and 126 ppb (μg/L) and bladder and lung cancer SMRs of 0, 81, 75, 96, and 114, respectively. The 18 villages that make up the low-dose group were from five townships. There were no villages from township 5 among the low-dose villages. Stratification of the bladder and lung cancer SMR analysis by township showed that the SMRs for townships 2, 4, and 6 were similar (and < 100) and that the SMRs for townships 0 and 3 were each significantly elevated (Figure 3).

Township-stratified linear regression analysis for bladder and lung cancer against median village well water arsenic level (micrograms per liter; ppb) for the low-dose villages yielded positive slopes (dose–response relationships) for townships 4 and 6 and negative slopes (dose–response relationships) for townships 0 and 3. Township 2 had only one village in the low-dose village group, and township 5 had none.

Thus, among the low-dose villages, townships 0 and 3 each showed a bladder and lung cancer mortality risk that was significantly increased over the background, greater than the risks in townships 2, 4, and 6 and independent of the median village well water arsenic exposure levels. The identity of this independent township factor is not known, although it may relate to the BFD prevalence township factor that Chen et al. (1985) had previously reported. Nonetheless, these analyses indicate that some geographically related risk factor exists in townships 0 and 3 that may be independent of arsenic exposure levels and appears to confound the dose–response analysis in the data set. As township 5 had no village among the low-dose villages and only two among the higher-dose villages, there were no data to examine risk at low dose in township 5.

The data for the 42 villages were separated into two groups—the data for townships 2, 4, and 6 that each showed a significant relationship to the median village well water arsenic level (*p* = 0.01–0.02) and the data for townships 0, 3, and 5 that showed some other possible nonarsenic carcinogenic risk factor and no apparent relationship to arsenic level [*p* = 0.24, 0.91, and undefined (based on only two data points), respectively].

**Figure 4:**
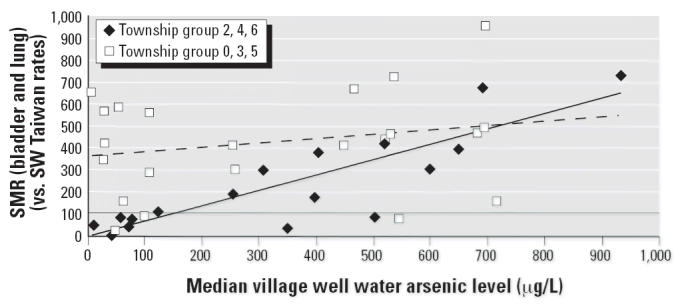
Bladder and lung cancer (combined) SMRs by township group and median village well water arsenic level (μg/L). Township group 0, 3, 5 (*n* = 22): *y* = 0.200*x* + 358; *R*^2^ = 0.053; *p* = 0.30. Township group 2, 4, 6 (*n* = 20): *y* = 0.704*x* + 6.26; *R*^2^ = 0.748; *p* = 0.001.

Figure 4 shows the dose–response relationship for bladder and lung cancer mortality risk and median village well water arsenic level by township group (townships 2, 4, and 6 vs. townships 0, 3, and 5) for the full set of 42 villages (range, 0.01–0.934 ppm). Median village well water arsenic level explains 75% of the variability in the bladder and lung cancer SMRs for the 20 villages in township group 2, 4, 6 (*p* < 0.001) and only 5% of that for the 22 villages in township group 0, 3, 5 (*p* = 0.30). The regression line for bladder and lung cancer SMR for township group 2, 4, 6 meets the no-increased-risk line (SMR = 100) at 151 μg/L with a 95% CI of 42 to 229 μg/L. The regression line for bladder and lung cancer SMR for township group 0, 3, 5 is above an SMR of 350 at 0 μg/L.

The regression line from bladder and lung cancer SMR for township group 2, 4, 6, meets the no-increased-risk line (SMR = 100) for males at 119 μg/L with a 95% CI of −70 to 229 μg/L and for females at 191 with a 95% CI of 66 to 280 μg/L.

Separate examination of the bladder cancer SMRs for the villages in township group 2, 4, 6 showed a significant dose–response relationship with median village well water arsenic level (*p* < 0.001) that meets the no-increased-risk line (SMR = 100) at 139 μg/L with a 95% CI of −7.5 to 233 μg/L. For the villages in township group 0, 3, 5, the dose–response relationship is not significant (*p* = 0.61), and the regression line is above SMR = 750 at 0 μg/L (data not shown). Likewise, examination of the lung cancer SMRs for the villages in township group 2, 4, 6 showed a significant dose–response relationship with median village well water arsenic level (*p* < 0.001) that meets the no-increased-risk line (SMR = 100) at 164 μg/L with a 95% CI of 26 to 257 μg/L. For the villages in township group 0, 3, 5, dose–response relationship is not significant (*p* = 0.16) and the regression line is above SMR = 250 at 0 μg/L (data not shown).

Further analysis was conducted among those townships (townships 2, 4, and 6) that appeared to show some relationship between cancer risk and arsenic exposure, eliminating those townships that appeared to have some other strong risk factor for bladder and lung cancer that was not related to the arsenic exposure (townships 0, 3, and 5).

**Figure 5:**
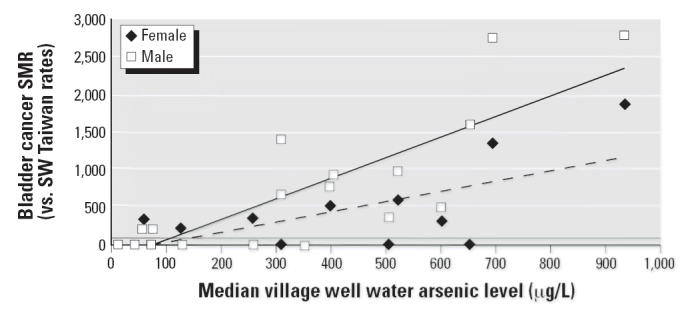
Bladder cancer SMRs for township group 2, 4, 6 for male and female by median village well water arsenic level (μg/L). Male bladder cancer: *y* = 2.75*x* + 243; *R*^2^ = 0.68; *p* < 0.001. Female bladder cancer: *y* = 1.33*x* + 117; *R*^2^ = 0.49; *p* = 0.001.

Figure 5 demonstrates, for male bladder cancer mortality in township group 2, 4, 6, a regression line (*p* < 0.001) that meets the no-increased-risk line (SMR = 100) at 125 μg/L with a 95% CI of −19 to 218 μg/L, and for female bladder cancer mortality, a regression line (*p* = 0.001) that meets the no-increased-risk line (SMR = 100) at 163 μg/L with a 95% CI of −80 to 294 μg/L.

**Figure 6:**
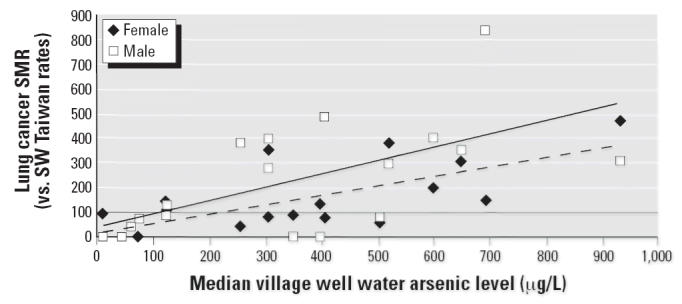
Lung cancer SMRs for township group 2, 4, 6 for male and female by median village well water arsenic level (μg/L). Male lung cancer: *y* = 0.536*x* + 37.5; *R*^2^ = 0.40; *p* = 0.003. Female lung cancer: *y* = 0.371*x* + 19.4; *R*^2^ = 0.52; *p* < 0.001.

Figure 6 demonstrates for male lung cancer mortality in township group 2, 4, 6, a regression line (*p* < 0.001) that meets the no-increased-risk line (SMR = 100) at 117 μg/L with a 95% CI of −274 to 273 μg/L, and for female lung cancer mortality, a regression line (*p* = 0.001) that meets the no-increased-risk line (SMR = 100) at 217 μg/L with 95% CI of 31 to 273 μg/L.

**Figure 7:**
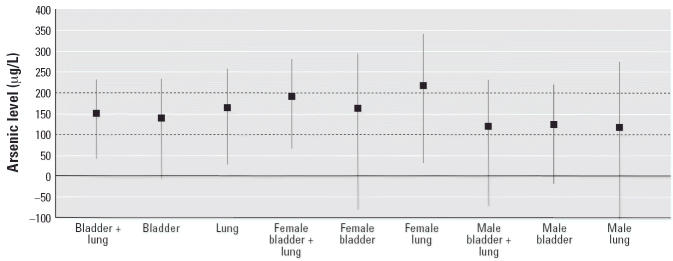
Exposure level (μg/L arsenic) at no-increased-risk level (SMR = 100) by cancer group for township group 2, 4, 6 (± 95% CI).

Figure 7 summarizes for township group 2, 4, 6 the exposure levels at which the regression line meets the no-increased-risk line (SMR = 100) with the 95% CI of that intercept in micrograms per liter arsenic. Most of these levels are in the range of 100–200 μg/L and statistically significantly different from zero overall. The fact that the intercept levels for females are greater than those for males and tend to be statistically significantly different from zero while those for the males do not, may reflect the effects of cigarette smoking, which is far less prevalent among the females than among the males.

## Discussion

Analysis of the bladder and lung cancer mortality data from the 42 study villages of the BFD-endemic area of SW Taiwan from the Wu et al. (1989) study showed only a 21% explanatory power when median village well water arsenic level is used as the sole explanatory variable in the linear regression model (*p* = 0.03). Separation of low-dose villages from higher-dose villages showed that the explanatory power resided among the higher-dose villages and did not appear among the low-dose villages. Previous analysis of the cancer mortality in the BFD-endemic had shown that township was a discriminating variable for the cancer mortality risk in this area (Chen et al. 1985). The mortality experience for the low-dose villages was examined to explore the contribution potentially made by township.

Within that analysis, certain townships (townships 0 and 3) were found each to have an increased cancer risk that was not related to arsenic exposure level, and other townships (townships 2, 4, and 6) were found to have cancer risks similar to each other with SMRs less than 100. The full 42-village data set was stratified by township group in order to examine the dose–response relationship in the absence of the interfering township factor. A significant positive dose response was seen with the data from townships 2, 4, and 6, with the explanatory power raised to 75% (*p* < 0.001). No significant positive dose response was seen with the data from townships 0, 3, and 5 (*p* = 0.30). Removal of the data from the townships influenced by the township factor markedly improved the fit of the data and the explanatory power of the model. If the SW Taiwan data are going to be used in formal risk analysis for ingested arsenic, the analysis should be restricted to the data from townships 2, 4, and 6.

**Figure 8:**
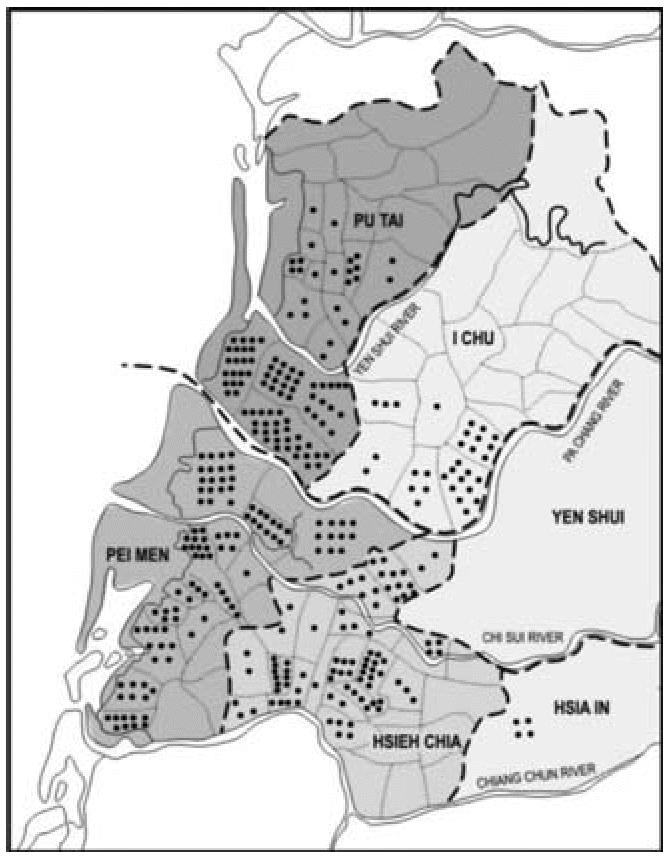
Distribution of BFD cases in the 1960s by township [adapted from Ch’i and Blackwell (1968) with permission of Oxford University Press].

The identity of this independent township factor is not known and may relate to the BFD prevalence township factor that Chen et al. (1985) had previously demonstrated. The data from Chen and Wu (1962) showed among the townships that the average number of wells per village, the proportion of villages with artesian well dependency, and the BFD prevalence were significantly correlated. Without knowing the specific identity and location of each study village, these various factors cannot be disentangled. Figure 8 shows that the BFD case distribution (Ch’i and Blackwell 1968) was not uniform over the study area. The number of BFD cases seems to be heavily concentrated in the three townships to the left of the figure.

The township factor may be a reflection of a selection bias occurring because the well water sampling was focused on the villages with high prevalence of BFD. Because the villages in the Wu et al. (1989) study were specifically selected because well water arsenic data from the 1960s existed for them, such a selection bias may have entered that could skew the interpretation of the results. High BFD prevalence was found in most but not all of the townships of Pei-men, Hsieh-chia, and Pu-tai; moderate prevalence, in the southern part of I-chu; and near absence in Hsia-in and Yen-shui. Again, it may not be possible to disentangle the relationship between BFD and any other possible etiologic factor for any outcome of interest, at least until each village can be specifically located on this map.

The “true” underlying structure of the arsenic dose–response relationship is more likely to be seen in the analysis of the cancer risks in townships 2, 4, and 6 than in that of the entire data set that contains the influence of the township factor. Removal of this extraneous source of variability has allowed for a clearer examination of the dose–response relationship. Interestingly, analysis of the bladder cancer mortality data for townships 2, 4, and 6 appears to display a fit to a threshold model. This finding for bladder cancer mortality is independently seen in males and females, suggesting that it may reflect a real phenomenon.

A thresholdlike model indicating that the bladder cancer mortality risk does not increase with exposure levels < 150 μg/L is consistent with other epidemiologic data. An ecologic analysis of the white male bladder cancer risk in the United States found no increase over an arsenic exposure range of 3–59 μg/L (Lamm et al. 2004). Case–control bladder cancer studies found no increased risk in the United States for exposures < 80 μg/day (Steinmaus et al. 2003) or < 160 μg/L (Bates et al. 1995) or in Argentina with exposures > 200 μg/L (Bates et al. 2004). The prospective cohort study in northeastern Taiwan reported that the multivariate-adjusted relative risk of urinary tract cancer was statistically significant for residents who drank well water containing arsenic at levels > 100 μg/L (Chiou et al. 2001). Cigarette smoking still remains a risk factor for bladder cancer.

A threshold, sublinear, or hormetic model for bladder cancer and inorganic arsenic exposure has been proposed from toxicologic studies, based on a number of modes of action (Snow et al. 2005). All three model forms have in common an inflection point wherein the risks at exposure levels greater than the inflection point do not predict the risks for exposures less than the inflection point exposure level. Suggested modes of action include generation of oxidative stress, perturbation of DNA methylation patterns, inhibition of DNA repair, and modulation of signal transduction pathways (Schoen et al. 2004). Cellular proliferation has been recognized as an early step in the development of bladder cancer by substances that are not mutagenic, such as inorganic arsenic (Cohen 2002).

A similar inflection point is seen in the analysis of the lung cancer mortality data and is statistically significant in females but not in males, although that question may not be answerable in the absence of smoking history. Perhaps analysis of lung cancer mortality in the northeastern Taiwan prospective study where smoking histories have been obtained would allow for a better assessment.

## Conclusion

Exploration of the SW Taiwan cancer mortality data set from the BFD-endemic area has identified significant confounding variables. Certain townships in the study demonstrate a significantly increased cancer risk at low-dose exposures that is independent of the village arsenic exposure levels. Removal of the data confounded by the township factor reveals an underlying dose–response curve for bladder and lung cancer mortality and arsenic level (median village well water arsenic level) that displays as a thresholdlike model. Such a model would not be contradicted by either the epidemiologic or the toxicologic literature, at least for bladder cancer.

The variability in the village SMRs, particularly the low-dose high-rate villages, is not explained by any biological model that uses arsenic as the sole explanatory variable. It is reasonable to assume that they are high for some nonarsenic reason. As Chen et al. (1985) demonstrated, the cancer mortality in the study area showed a dose–response relationship with BFD prevalence at both a village and township level of analysis. We have followed through from these findings and have analyzed separately the data from the townships that do not appear to have been strongly influenced by this second factor that may be related to BFD prevalence. It is likely that the cancer dose–response relationship is more clearly seen in those areas that have little or no confounding by BFD prevalence.

## Correction

The following figures have been modified from the originally published article online:

Figure 1: Now includes the horizontal regression line at SMR = 100 to indicate the level at which no increased risk is observed.

Figure 3: Now includes township group 0, 3, 5 (Twn grp 0, 3, 5). A vertical line has been added to separate the township-specific results within each township, and the 95% CI values have been added to the Twn 2 data point.

Figure 7: 95% CI values have been corrected for female bladder and female lung cancers.

## Figures and Tables

**Table 1:** Internal cancer data from arsenic-exposure studies conducted in Taiwan region endemic to BFD (corrected).

					Person-years		Bladder		Lung		Liver	
	Village	No. of wells	Arsenic concentration (ppm)	Median	M	F	M	F	M	F	M	F
1	3-H	1	0.010	0.010	4,159	4,043	1	6	3	5	3	1
2	2-I	1	0.011	0.011	3,529	3,194	0	0	0	1	0	0
3	0-G	5	0.010, 0.010, 0.030, 0.259, 0.770	0.030	5,388	4,861	3	2	4	5	3	3
4	3-5	1	0.032	0.032	7,851	7,033	3	3	6	2	5	2
5	3-N	1	0.032	0.032	2,689	2,392	4	3	3	1	1	1
6	4-7	1	0.042	0.042	10,629	10,227	0	0	0	0	4	0
7	6-A	1	0.045	0.045	7,716	6,820	0	0	0	0	1	1
8	0-J	2	0.020, 0.080	0.050	6,501	5,888	1	0	0	0	2	2
9	3-L	2	0.053, 0.058	0.056	6,238	5,094	3	4	5	7	3	0
10	4-D	1	0.060	0.060	10,107	9,227	1	2	1	1	1	1
11	3-P	1	0.065	0.065	6,574	5,927	0	0	2	5	3	0
12	6-C	1	0.073	0.073	12,767	11,937	0	1	2	0	2	0
13	4-8	1	0.080	0.080	11,307	10,332	1	0	2	2	3	1
14	O-O	1	0.100	0.100	6,895	6,392	0	0	3	1	2	2
15	O-E	5	0.010, 0.085, 0.110, 0.288, 0.686	0.110	5,753	5,310	6	3	4	5	3	1
16	O-I	7	0.020, 0.050, 0.110, 0.110, 0.190, 0.580, 0.590	0.110	4,249	3,833	0	2	3	2	1	3
17	4-N	2	0.073, 0.172	0.123	4,709	4,291	0	0	1	2	3	1
18	4-J	1	0.126	0.126	6,508	6,026	0	1	2	2	6	1
19	2-D	1	0.256	0.256	9,702	8,869	0	2	7	1	2	1
20	O-D	1	0.256	0.256	3,872	3,412	1	3	5	2	2	3
21	3-Q	6	0.148, 0.198, 0.242, 0.276, 0.291, 0.458	0.259	5,580	5,079	2	0	5	4	4	2
22	4-M	1	0.307	0.307	2,953	2,758	1	0	2	3	0	0
23	6-6	1	0.307	0.307	5,364	4,505	3	0	4	1	3	1
24	4-E	2	0.340, 0.360	0.350	3,912	3,586	0	0	0	1	1	0
25	4-L	2	0.310, 0.485	0.398	3,069	2,723	1	1	0	1	1	0
26	4-F	11	0.120, 0.170, 0.229, 0.260, 0.260, 0.406, 0.469, 0.485, 0.595, 0.779, 0.819	0.406	4,482	3,886	2	3	5	1	1	0
27	3-I	1	0.448	0.448	4,551	4,259	2	3	4	3	5	1
28	5-G	1	0.467	0.467	6,179	5,298	7	5	7	1	2	3
29	4-P	1	0.504	0.504	5,843	5,397	1	0	1	1	1	1
30	0-H	5	0.050, 0.394, 0.520, 0.610, 1.752	0.520	4,390	4,313	3	2	4	5	4	0
31	4-I	47	0.020, 0.020, 0.030, 0.090, 0.100, 0.110, 0.120, 0.120, 0.160, 0.190, 0.230, 0.240, 0.250, 0.270, 0.270, 0.290, 0.290, 0.350, 0.370, 0.410, 0.430, 0.450, 0.510, 0.520, 0.540, 0.560, 0.660, 0.700, 0.730, 0.740, 0.760, 0.760, 0.760, 0.780, 0.810, 0.810, 0.840, 0.840, 0.850, 0.850, 0.850, 0.870, 0.890, 0.900, 0.930, 0.940, 0.970	0.520	4,870	4,432	2	2	3	5	1	0
32	3-J	2	0.529, 0.529	0.529	9,454	8,689	4	8	6	5	3	1
33	3-S	2	0.480, 0.595	0.538	4,287	3,667	4	3	8	4	7	0
34	3-9	1	0.544	0.544	3,655	3,413	0	1	1	0	1	1
35	2-2	10	0.560, 0.580, 0.580, 0.590, 0.597, 0.600, 0.618, 0.620, 0.650, 0.704	0.599	9,059	7,977	2	2	8	5	9	5
36	4-G	2	0.620, 0.680	0.650	2,425	2,108	2	0	2	2	0	0
37	5-4	2	0.630, 0.735	0.683	3,155	2,983	1	1	5	2	2	1
38	2-M	2	0.435, 0.950	0.693	11,123	11,263	9	9	14	4	6	4
39	0-F	5	0.415, 0.660, 0.694, 0.720, 0.749	0.694	7,010	5,720	5	1	2	9	8	3
40	3-R	5	0.397, 0.440, 0.698, 0.750, 1.010	0.698	4,310	3,576	3	6	6	7	3	2
41	3-M	4	0.221, 0.329, 1.105, 1.411	0.717	5,815	4,877	0	1	0	4	2	0
42	2-N	3	0.560, 0.934, 0.960	0.934	8,341	8,342	7	10	4	10	8	2
Total	153			256,970	233,959	85	90	144	122	122	51

Abbreviations: F, female; M, male.

Data from Wu et al. (1989) and Chen et al. (1992). Table from NRC (1999b), reprinted with corrections with permission from the National Academy of Sciences, courtesy of the National Academies Press. See Supplemental Material for original corrected table with comments (http://www.ehponline.org/members/2006/8704/suppl.pdf)]. For raw data, see the StatLib website hosted by Carnegie Mellon University (Carnegie Mellon University 2006); click on “Get Data,” then search the term “arsenic.”
