# Supplementary material for: Arsenic Cancer Risk Confounder in Southwest Taiwan Data Set
Source: Environ Health Perspect. 2006 Jan 13;114(7):1077–82. doi: 10.1289/ehp.8704 (PMC1513326; doi:10.1289/ehp.8704)
Supplement: Supplemental Figures and Tables [file ehp0114-001077s1.pdf]

June 1, 2006 Version of NRC Table (corrected)

Supplemental Data

Below is a corrected version of a table originally published by the National Academy Press [National Research Council, 1999. Internal Cancer Data from Arsenic-Exposure Studies in Taiwan Region Endemic to Blackfoot Disease. Addendum to Chapter 10. Table A 10-1. In: Arsenic in Drinking Water. Washington, DC: National Academy Press, 308-309]. The original publication had the data for lung and liver cancers transposed and had incorrect data for female lung cancers in villages 4-D, 3-M, and 2-N and for male person-years in villages 4-E, 5-G, and 3-J. Village numbers and totals have been added.

Table A 10-1 Internal Cancer Data from Arsenic-Exposure Studies Conducted in Taiwan Region Endemic to Blackfoot Disease

		Number			Person-Years		Bladder		Lung		Liver	
	Village	of Wells	Arsenic Concentration, ppm	Median	M	F	M	F	M	F	M	F
1	3-H	1	0.010	0.010	4,159	4,043	1	6	3	5	3	1
2	2-I	1	0.011	0.011	3,529	3,194	0	0	0	1	0	0
3	0-G	5	0.010, 0.010, 0.030, 0.259, 0.770	0.030	5,388	4,861	3	2	4	5	3	3
4	3-5	1	0.032	0.032	7,851	7,033	3	3	6	2	5	2
5	3-N	1	0.032	0.032	2,689	2,392	4	3	3	1	1	1
6	4-7	1	0.042	0.042	10,629	10,227	0	0	0	0	4	0
7	6-A	1	0.045	0.045	7,716	6,820	0	0	0	0	1	1
8	0-J	2	0.020, 0.080	0.050	6,501	5,888	1	0	0	0	2	2
9	3-L	2	0.053, 0.058	0.056	6,238	5,094	3	4	5	7	3	0
10	4-D	1	0.060	0.060	10,107	9,227	1	2	1	1	1	1
11	3-P	1	0.065	0.065	6,574	5,927	0	0	2	5	3	0
12	6-C	1	0.073	0.073	12,767	11,937	0	1	2	0	2	0
13	4-8	1	0.080	0.080	11,307	10,332	1	0	2	2	3	1
14	O-O	1	0.100	0.100	6,895	6,392	0	0	3	1	2	2
15	O-E	5	0.010, 0.085, 0.110, 0.288, 0.686	0.110	5,753	5,310	6	3	4	5	3	1
16	O-I	7	0.020, 0.050, 0.110, 0.110, 0.190, 0.580, 0.590	0.110	4,249	3,833	0	2	3	2	1	3
17	4-N	2	0.073, 0.172	0.123	4,709	4,291	0	0	1	2	3	1
18	4-J	1	0.126	0.126	6,508	6,026	0	1	2	2	6	1
19	2-D	1	0.256	0.256	9,702	8,869	0	2	7	1	2	1
20	O-D	1	0.256	0.256	3,872	3,412	1	3	5	2	2	3
21	3-Q	6	0.148, 0.198, 0.242, 0.276, 0.291, 0.458	0.259	5,580	5,079	2	0	5	4	4	2
22	4-M	1	0.307	0.307	2,953	2,758	1	0	2	3	0	0
23	6-6	1	0.307	0.307	5,364	4,505	3	0	4	1	3	1
24	4-E	2	0.340, 0.360	0.350	3,912	3,586	0	0	0	1	1	0
25	4-L	2	0.310, 0.485	0.398	3,069	2,723	1	1	0	1	1	0
26	4-F	11	0.120,0.170, 0.229, 0.260, 0.260, 0.406, 0.469, 0.485, 0.595, 0.779, 0.819	0.406	4,482	3,886	2	3	5	1	1	0
27	3-I	1	0.448	0.448	4,551	4,259	2	3	4	3	5	1
28	5-G	1	0.467	0.467	6,179	5,298	7	5	7	1	2	3
29	4-P	1	0.504	0.504	5,843	5,397	1	0	1	1	1	1
30	0-H	5	0.050, 0.394, 0.520, 0.610, 1.752	0.520	4,390	4,313	3	2	4	5	4	0
31	4-I	47	0.020, 0.020, 0.030, 0.090, 0.100, 0.110, 0.120, 0.120, 0.160, 0.190, 0.230, 0.240, 0.250, 0.270, 0.270, 0.290, 0.290, 0.350, 0.370, 0.410, 0.430, 0.450, 0.510, 0.520, 0.540, 0.560, 0.660, 0.700, 0.730, 0.740, 0.760, 0.760, 0.760, 0.780, 0.810, 0.810, 0.840, 0.840, 0.850, 0.850, 0.850, 0.870, 0.890, 0.900, 0.930, 0.940, 0.970	0.520	4,870	4,432	2	2	3	5	1	0
32	3-J	2	0.529, 0.529	0.529	9,454	8,689	4	8	6	5	3	1
33	3-S	2	0.480, 0.595	0.538	4,287	3,667	4	3	8	4	7	0
34	3-9	1	0.544	0.544	3,655	3,413	0	1	1	0	1	1
35	2-2	10	0.560, 0.580, 0.580, 0.590, 0.597, 0.600, 0.618, 0.620, 0.650, 0.704	0.599	9,059	7,977	2	2	8	5	9	5
36	4-G	2	0.620, 0.680	0.650	2,425	2,108	2	0	2	2	0	0
37	5-4	2	0.630, 0.735	0.683	3,155	2,983	1	1	5	2	2	1
38	2-M	2	0.435, 0.950	0.693	11,123	11,263	9	9	14	4	6	4
39	0-F	5	0.415, 0.660, 0.694, 0.720, 0.749	0.694	7,010	5,720	5	1	2	9	8	3
40	3-R	5	0.397, 0.440, 0.698, 0.750, 1.010	0.698	4,310	3,576	3	6	6	7	3	2
41	3-M	4	0.221, 0.329, 1.105, 1.411	0.717	5,815	4,877	0	1	0	4	2	0
42	2-N	3	0.560, 0.934, 0.960	0.934	8,341	8,342	7	10	4	10	8	2
	Total	153			256,970	233,959	85	90	144	122	122	51

Data from Wu et al. 1989; Chen et al. 1992

Source: National Research Council (1999). Arsenic in Drinking Water. Washington, DC: National Academy Press, p 308-09 (corrected).
